# Exoscope-assisted temporal bone resection: operative videos of the lateral and total techniques

**DOI:** 10.3171/2023.10.FOCVID23135

**Published:** 2024-01-01

**Authors:** Alejandra Rodas, Edoardo Porto, Juan M. Revuelta Barbero, Jackson R. Vuncannon, Youssef M. Zohdy, Biren Khimji Patel, Gustavo Pradilla, C. Arturo Solares

**Affiliations:** 1 Department of Otolaryngology and; 2 Department of Neurosurgery, Emory University, Atlanta, Georgia

**Keywords:** exoscope, total temporal bone resection, lateral temporal bone resection

## Abstract

Skull base malignancies arising from the parotid gland, skin, or external auditory canal (EAC) can potentially involve the temporal bone. Management of these invasive tumors represents a true challenge considering the critical neurovascular relationships. Exoscope-assisted temporal bone resection (TBR) plays a crucial role in addressing such malignancies. The extent of disease is evaluated using the Pittsburgh staging system, which then guides the boundaries of resection. Lateral TBR (LTBR) relies on removal of the EAC and lateral ossicles and is generally appropriate for stage T1 and T2 tumors. Total TBR (TTBR) is reserved for high-grade tumors involving the petrous apex.

The video can be found here: https://stream.cadmore.media/r10.3171/2023.10.FOCVID23135

## Transcript

Exoscope-assisted temporal bone resection: operative videos of the lateral and total techniques.

### 0:30 Case 1: Clinical History.

The patient is a 79-year-old male who presented with 1-month history of right-sided otorrhea, odor, and hearing loss. Debris and polyps were observed on examination. The external auditory canal lesion was biopsied, and the results evidenced a well-differentiated squamous cell carcinoma with invasion into the underlying dermis. Twelve years prior to presentation, the patient had a T1N1 right glossotonsillar squamous cell carcinoma for which he underwent a right tonsillectomy, neck dissection, and radiotherapy.

### 1:01 Case 1: Preoperative Imaging.

Preoperative CT showed a focal lesion centered within the bony component of the external auditory canal measuring 1.1 × 1.1 cm. There was slight erosion of the bony portion of the external auditory canal and right mastoid effusion. The inner ear appeared intact.

### 1:21 Surgical Setup.

Aligning the screen parallel with the surgeon’s eyes allows a more neutral position with precise control of the surgical field. The ergonomic changes offered by the exoscope have been reported with higher preference rates when compared with the use of microscopes.^1^

### 1:32 Surgical Setup.

The exoscope’s optical system does not interfere with the surgeon’s space and instrument mobilization, as it is placed approximately 30 cm away from the surgical field. The 3D monitor is positioned at the level of the surgeon’s eyes, across the operating table, reducing the neck strain and shoulder tension. The exoscope, compared to the binocular operating microscope, allows greater magnification, a wider focal distance, better surgeon ergonomics, and noninferior focal and field depth perception. In addition, the same vision is provided to the surgeons and the operating room staff in general.^1^

### 2:10 Positioning and Exposure.

The patient is positioned supine with the head turned, facing the side opposite to the lesion. The tip of the mastoid is the most superior landmark in the field. A C-shaped postauricular incision is performed, with parietal scalp and cervical extensions to allow temporalis flap harvest and possible neck dissection, respectively.

### 2:28 Case 1: Lateral Temporal Bone Resection Dissection.

Based on the patient’s Pittsburgh stage II squamous cell carcinoma, a right lateral temporal bone resection was performed, along with a superficial parotidectomy. After a circumferential incision around the right external acoustic meatus and preauricular incision to the level of the parotideomasseteric fascia, the cartilaginous external auditory canal was severed and the mastoid portion of the temporal bone was fully exposed along with the root of the zygoma, spine of Henle, and tympanic bone.

### 2:58 Case 1: Surgical Video.

At this point, the exoscope was brought into the field for high-power magnification and microdissection. A complete mastoidectomy was performed, identifying the tegmen tympani superiorly and the sigmoid sinus posteriorly. The incus was identified in the antrum. Drilling was then extended anteriorly in the epitympanum until the soft tissue of the glenoid fossa was encountered. The mastoid segment of the facial nerve was then identified and skeletonized from the second genu to the stylomastoid foramen. A posterior tympanostomy was developed and the incus buttress drilled away. The incustapedial joint was severed with a tab knife and the incus removed from the mesotympanum. The posterior tympanotomy was extended to the hypotympanum, sacrificing the chorda tympani in the process. The hypotympanic cut was extended to the temporomandibular joint capsule and carried laterally through the lateral aspect of the mastoid process. At this point, the external auditory canal was attached only anteriorly at the tympanic plate. The tensor tympani was severed with a Bellucci scissor. Gentle pressure on the posterior aspect of the external auditory canal fractured the tympanic plate, freeing the lateral temporal bone resection. The resection cavity was then copiously irrigated and the eustachian tube was packed with a piece of sternocleidomastoid fascia and Surgicel. With facial nerve stimulation, full facial movement was noted.

### 4:29 Case 1: Postoperative Imaging.

Postoperative imaging revealed postsurgical changes from partial right mastoidectomy and temporalis myofascial flap reconstruction. No residual disease was observed.

### 4:41 Case 1: Postoperative Outcomes.

Pathology confirmed the previous diagnosis, reporting a well-differentiated squamous cell carcinoma with negative perineural invasion and negative lymphovascular invasion. Free surgical margins were also reported. There was no evidence of disease after 1 year of follow-up. There was adequate integration and vascularization of the temporalis muscle flap used for reconstruction.

### 5:05 Case 2: Clinical History.

The patient is a 67-year-old male with history of left-sided otalgia, hearing loss, and aural fullness. Nine months prior to his current presentation, the patient had a stage 4 anterior skull base mass reported as intestinal type sinonasal adenocarcinoma. At that point, the patient was treated with endoscopic craniofacial resection and radiotherapy.

### 5:26 Case 2: Preoperative Imaging.

Preoperative MRI scans revealed a lesion completely occupying the middle ear cavity and left mastoid air cells, with extension into the petrous apex. Postsurgical changes secondary to complete bilateral ethmoidectomies and sphenoidotomy are also appreciated.

### 5:43 Case 2: Total Temporal Bone Resection—Dissection and Surgical Video.

After a C-shaped postauricular incision, the middle cranial fossa was accessed via a temporal craniotomy and subtemporal approach. At the middle cranial fossa floor, the arcuate eminence, foramen spinosum, and trigeminal nerve were observed. The mastoid, zygoma, and mandible were uncovered, and osteotomies improved visualization of the infratemporal fossa as the mandibular ramus and styloid process were released. Upon locating the carotid canal, the vertical segment of the internal carotid artery was revealed. Subsequently, the dura mater of the posterior fossa and the sigmoid sinus were gently separated from the posterior petrous surface. The carotid sheath within the infratemporal fossa was used to locate and trace the internal carotid artery and internal jugular vein. After identification and drilling of the internal auditory canal in the petrous bone, cranial nerves VII and VIII were sacrificed as they entered the canal. Once the temporal bone had been accessed and freed from nearly all directions, it remained attached medially at the level of the petrous apex. The procedure finalized with an en bloc resection of the temporal bone.

### 7:54 Case 2: Reconstruction and Closure.

Dural tears were repaired primarily. A temporalis muscular flap was rotated posteriorly into the surgical defect to protect the petrous carotid artery and the jugular bulb as well as buttress the internal acoustic canal dural repair. The patient then underwent a left anterolateral thigh free tissue transfer to the surgical defect, which was anastomosed to the external carotid system and common facial vein. The anteriorly based skin flap was returned to its native position. The external auditory canal was closed in a blind pouch.

### 8:26 Case 2: Postoperative Imaging.

Postoperative imaging revealed postsurgical changes, including temporal bone and lateral orbital wall resection. The reconstructive flap is also noted overlying the cranial defect.

### 8:38 Case 2: Postoperative Outcomes.

The final pathology revealed a 1.8-cm intestinal type adenocarcinoma. Negative margins were achieved and lymphovascular and perineural invasion were both negative. The anterolateral thigh flap used for reconstruction proved to be viable. The left cranial nerves VII and VIII were transected during surgery at the level of the internal auditory canal. Facial paralysis was noted postoperatively. Complaints of dizziness and imbalance were also reported. Three years after temporal bone resection, the patient presented with a sphenoclival mass consistent with adenocarcinoma. Further endoscopic resection was selected as treatment for recurrence.

### 9:17 Lateral Temporal Bone Resection.

Lateral temporal bone resection surgery is indicated for lesions at the bony external auditory canal, essentially lateral to the tympanic membrane. It has been reported to be the modality of choice for temporal bone tumors with Pittsburgh stage T1 or T2.^2^ This technique results in loss of conductive hearing capacity as the external auditory canal is resected en bloc along with the tympanic membrane and lateral ossicles.^3^ A mastoidectomy is performed and the facial recess is exposed. The procedure may be supplemented by parotidectomy and neck dissection.^4^

### 9:50 Total Temporal Bone Resection.

Temporal bone tumors with Pittsburgh stage T4 are treated with total temporal bone resection. These tumors include those that erode the cochlea, petrous apex, medial wall of the middle ear, carotid canal, jugular foramen, or dura.^2^ Total temporal bone resection involves resection of the petrous apex. The infratemporal fossa is dissected, requiring a segmental mandibulectomy and zygomatic arch osteotomies. The petrous segment of the ICA may be preserved.^5^

## Disclosures

Dr. Solares reported personal fees from Stryker outside the submitted work.

## Author Contributions

Primary surgeon: Solares, Pradilla. Assistant surgeon: Vuncannon. Editing and drafting the video and abstract: Solares, Rodas, Porto, Revuelta Barbero, Vuncannon, Patel. Critically revising the work: Solares, Rodas, Porto, Revuelta Barbero, Vuncannon, Zohdy, Pradilla. Reviewed submitted version of the work: Rodas, Porto, Revuelta Barbero, Vuncannon, Zohdy, Pradilla. Approved the final version of the work on behalf of all authors: Solares. Supervision: Solares, Rodas, Porto, Revuelta Barbero, Pradilla. Narration: Rodas.

## Supplemental Information

### Patient Informed Consent

The necessary patient informed consent was obtained in this study.
